# Effect of kidney transplantation on right ventricular function, assessment by 2- dimensional speckle tracking echocardiography

**DOI:** 10.1186/s12947-020-00200-7

**Published:** 2020-05-26

**Authors:** Mohammad Khani, Ahmad Tara, Shadi Shekarkhar, Morteza Abdar Esfahani, Fariba Bayat

**Affiliations:** 1 Cardiovascular Research Center Shahid Beheshti University of Medical Sciences, Po.Box: 1998734383, Tehran, Iran; 2 Faculty of Medicine, Department of Cardiology & echocardiography, Isfahan University of medical science, Isfahan, Iran

**Keywords:** 2-D speckle tracking echocardiography, Kidney transplant

## Abstract

**Background:**

Advanced chronic kidney disease often results in adverse cardiovascular outcomes and is the leading cause of mortality in patients with end stage renal diseases (ESRD). There is much information about the effect of chronic kidney diseases (CKD) on the left ventricle (LV) chamber, but the right ventricle (RV) as a neglected chamber had not been evaluated precisely, in spite of its importance.

**Objectives:**

The aim of this study was to evaluate the impact of successful kidney transplants on the RV systolic and diastolic function using the advanced method of 2D speckle tracking echocardiography and comparison with the conventional methods.

**Method:**

The study included 48 patients with CKD who were eligible for kidney transplantation and underwent successful kidney transplantations. Right ventricular indices were evaluated, while RV function was focused by conventional methods and 2D speckle tracking echocardiography before the successful kidney transplant and 1 week, 1 month, and 3 months after the successful kidney transplant.

**Results:**

The results of the study showed that RV global longitudinal strain and RV free wall longitudinal strain improved over the time (*P* = 0.024, *P* < 0.001 respectively). It also represented that kidney transplantation did not have significant effect on the RV mid cavity diameter, tissue velocity, Myocardial performance index, RV longitudinal diameter, and Tricuspid annular plane systolic excursion indices, but for other indices this effect was significant. On the differences between the mean slope of regression line of the GLS variable in hypertensive subjects (1.0 ± 0.2) and non-hypertensive subjects (0.36 ± 0.32), an independent t-test showed that between the two groups in terms of the improvement of RVGLS, there was a significant statistical difference (*P* = 0.0067).

**Conclusion:**

Most of the ESRD patients had subtle RV dysfunction which could be better detected by recent echocardiography methods than conventional methods. Moreover, kidney transplantation led to considerable improvements in RV function in this population.

## Introduction

Accounting for 40% of deaths in international registries, cardiac disease is the main cause of death in dialysis patients [1]. It is due to progressive cardiorenal compromise which causes adverse cardiovascular outcomes not only by accelerated atherosclerosis and subsequent coronary artery disease, but also by other mechanisms such as hemodynamic overload from volume and pressure, anemia, arteriovenous shunts and arterial remodeling as well as biochemical mediators and uremic toxics [2].

Kidney transplant in comparison to dialysis lessens morbidity and mortality [3], applying better surgical techniques and immunosuppression therapy. Nowadays, kidney transplant is considered as the standard treatment for patients with ESRD [4].

Echocardiography is a tool for the noninvasive assessment of hemodynamic parameters and the evaluation of cardiac chamber size and function of the heart. Recently, speckle tracking echocardiography (STE) has been considered as an advanced method that can evaluate subtle myocardium diseases.

Data about the effect of CKD on the LV chamber are extensive, but the RV as a neglected chamber is not evaluated precisely, in spite of its importance. Two-dimensional STE has been validated as a promising tool for the evaluation of RV systolic function in several clinical settings [5].

The aim of the present study was to evaluate the effect of kidney transplant on the heart using two-dimensional speckle tracking echocardiography and conventional methods, focusing on the RV size and function.

### RV function

Having an intricate anatomic structure, the RV is divided into two parts including the inflow and outflow tracts which are separated by crista supraventricularis.

The form and motion pattern of the RV are intricate and there are problems with defining these endocardial surface of the thin RV free wall, so echocardiographic imaging of RV and measuring the ejection fraction have been tricky. The changeable loading conditions factor is regarded as the other important issue complicating the interpretation of RV function. Traditionally, RV function has been qualitatively assessed by 2D echocardiography.

The systolic contraction of RV lateral wall leads to about 27% longitudinal shortening, while the base descends forward the peak which is pretty stationary [6, 7]. Besides, circumferential shortening constricting the ventricle can be observed. The interventricular septum contributes to RV and LV function and the relatively contribution can be different in the normal and the diseased heart .

The RV fractional area change usually applied as a measure of RV function can be considered as an area-based measure of RV function being calculated from a four-chamber view [8]. A generally used measure of RV function is Tricuspid Annular Plane systolic excursion (TAPSE) which is measured by M-mode echocardiography. Generally, TAPSE is bigger than 2 cm [8]. TAPSE associates with RV ejection fraction which is measured by radionuclide angiography [9, 10] PASP can be estimated using TR velocity. RVSP can be reliably determined from peak TR jet velocity,using the simplified bernoulli equation and combining this value with an estimated of RA pressure: RVSP = 4(V)^2^ + RA pressure, where V is the peak velocity (in meters per seconds) of tricuspid valve regurgitant jet, and RA pressure is estimated from IVC diameter and respiratory changes (5mmhg was added in all patients with IVC size≤2 cm and collapsibility> 50%,10 mmhg in those with IVC > 2 cm or collapsibility< 50% and 15–20 mmhg in others as RA pressure) [11] (Table 1).

**Table 1:** The prevalence and changes of IVC size and respiratory collapsibility before and after kidney transplant over the time

	Before transplant	One week after transplant	One month after transplant	3 months after transplant	Mean square	F	*P*-value
IVC < 21 mm	83.3%	87.5%	89.6%	92.9%	0.085	0.851	0.451
Respiratory collapse≥50%	91.7%	95.8%	91.7%	97.6%	0.849	0.480	0.508

The Tissue Doppler imaging (TDI) measurement of displacement can give a similar measure. Global RV strain is the mean peak systolic strain from the three RV lateral wall segments [7, 12]. Being compared with the free lateral wall, the septal segments enjoy a smaller amount of deformations, so this makes the interpretation of the results difficult.

A condensed global RV strain expected worse prognosis in numerous diseases having an effect on the right ventricle such as CKD; therefore, the current study was an attempt to demonstrate that kidney transplant could cause reverse remolding of RV which may be insignificant and invaluable by conventional tools of measurement but may be more obvious and significant by 2D speckle tracking echocardiography.

## Method

### Study population

The current study was a longitudinal, prospective one being performed to evaluate echocardiography findings of 48 patients including 28 males (58.3%) and 20 females (41.7%) with ESRD undergoing living-donor kidney transplantation in four steps (i.e., before kidney transplant and 1 week, 1 month, and 3 months after kidney transplant) at Shahid Modarres Hospital, Tehran, Iran, from January 2017 to January 2018. All the patients were in a stable condition and were carefully reviewed by a committee to be eligible for kidney transplantation after evaluating risks and benefits. Basic and demographic data of the participants according to their gender were summarized in Table 2.

**Table 2:** Basic and demographic data of the participants according to their gender

Variables	Category	Male (*n* = 28)	Female (*n* = 20)	Total population Mean (SD)/n. (%)	*P*-value^1^
		Mean (SD)/num (%)	Mean (SD)/num (%)		
Age	Year	43.8 ± 14	42.6 ± 14.1	43.5 ± 13.9	0.79
BMI	(kg/m^2^)	23.7 ± 3.9	26.1 ± 7.3	24.7 ± 5.7	0.13
Phosphor		5.71 ± 1.41	5.95 ± 1.29	5.81 ± 1.35	0.56
Creatinine		8.18 ± 3.0	7.71 ± 2.67	7.99 ± 2.88	0.57
Calcium		8.77 ± 1.0	7.9 ± 1.15	8.42 ± 1.17	0.012
Ferritin		61.6 ± 34.4	49.6 ± 25.8	56.6 ± 31.4	0.19
Urea		113.5 ± 40.1	115.9 ± 41.6	114.5 ± 40.3	0.84
Hemoglobin		11 ± 2.0	10.1 ± 2.0	10.6 ± 2.0	0.16
Duration of dialysis	pre-emptive	3 (10.7)	3 (15)	6 (12.5)	0.48
	< 6 months	4 (14.3)	6 (30)	10 (20.83)	
	6–12 months	6 (21.4)	4 (20)	10 (20.83)	
	> 12 months	15 (53.6)	7 (35)	20 (45.83)	
Dialysis type	pre-emptive	3 (10.7)	3 (15)	6 (12.5)	0.55
	Hemodialysis	21 (75)	16 (80)	37 (77.08)	
	Peritoneal dialysis	4 (14.3)	1 (5)	5 (10.42)	
Diabetes	Yes	6 (21.4)	5 (25)	11 (22.92)	0.77
Hypertension	Yes	19 (70.4)	12 (60)	31 (65.96)	0.45
Smoking	Yes	4 (14.3)	0 (0)	4 (8.33)	0.07

^1^value is presented as mean ± SD or n. (%) compared using independent T-test or chi square test, respectively

All the recipients received a standard protocol with the post-transplant medical regimen including tacrolimus, mycophenolate mofetil, and prednisolone.

Among these 48 ESRD cases, regard to type of replacement therapy before transplantation, some of them were pre-emptive kidney transplantation [13] (refers to transplantation before initiation of chronic maintenance dialysis), some of them underwent peritoneal dialysis, and the other major were treated with hemodialysis.

We excluded the recipients of kidney transplants from cadaver and the patients underwent emergent transplantations due to any reasons because we could not gather their pre-operation echocardiographic information.

The participants’ demographic, echocardiographic, and laboratory data are presented below.

### Echocardiography

All echocardiographic studies were performed by IE 33 (Philips medical system, USA) and 2D echocardiographic imaging using an S4 transducer.

We acquired the apical RV view to measure different indices of RV size and function, and then the off-line analysis of recorded images was done using CMQ Q-lab 9 (Philips medical system, USA). Images were obtained with the patient in the left lateral decubitus position.

An experienced echocardiographer obtained all the images using the standard protocol. Then, one of the researchers, who were blind to the clinical data of the patients and study, analyzed the data and we requested another expert echocardiographer to analyze the strain imaging offline (measurement of already acquired data and the observer constrained by measuring the same cardiac cycles) intra-observer and inter-observer variability were assessed for strain analysis which were 0.92(0.90–0.94) and 0.90(0.87–0.93) respectively.

The goal of the current study was to evaluate the RV function using different indices and especially by 2D STE. We measured the LV size, LVEDV,LV CO, and LVEF and also LA volume and indexed them base on Body Surface Area in 2D and assessed the LV diastolic function by TDI according to Lang et al. [14] and summarized the data in Table 3. for the RV chamber, we measured the RV size at the base, mid, and length and the RVOT size in the proximal and distal part of SAX and parasternal long axis and also the RA size in major and minor dimensions and end systolic area of RA according to Rudski et al. [11].

**Table 3:** Changes of recommended measures of left ventricle in 48 studied participants over the time

LV function indices	Before transplant	One week after transplant	One month after transplant	3 months after transplant	Mean square	F	*P*-value
LA volume index	31.27 ± 5.5	30.89 ± 4.6	29.27 ± 3.5	26.69 ± 3.6	200.31	15.2	0.000
LVEDV	96.8 ± 12	102.18 ± 13	103.95 ± 9.8	96.54 ± 8.8	804.79	6.4	0.001
LVEF	58.53 ± 5.7	56.19 ± 7.3	57 ± 7.3	58.5 ± 4.3	42.12	1.79	0.166
CO/index	3.05 ± 48	3.22 ± 0.54	3.5 ± 0.40	3.44 ± 0.47	1.59	6.25	0.001
E/e	0.78 ± 0.20	0.76 ± 0.20	0.86 ± 0.22	0.92 ± 0.24	0.241	5.94	0.002

We evaluated the RV function by TAPSE, FAC, S velocity, MPI all according to Rudski et al. as explain here: M-mode was employed to assess the TAPSE. The M-mode cursor was placed through the lateral side of the tricuspid annulus in such a way that the position of annulus, changed along with the cursor. The systolic displacement of annular was assessed from end-diastole to end-systole. The *tricuspid annular systolic velocity* (s′) was measured in the apical 4-chamber view though tissue Doppler imaging. The *isovolumic acceleration of the RV* was computed as the peak isovolumic myocardial velocity divided by the time to peak velocity, evaluated by tissue Doppler imaging at the lateral tricuspid annulus. The *RV Fractional Area Change* was calculated by this formula RVFAC = (RV diastolic area–RV systolic area)/RV diastolic area  ×  100%. The RV diastolic and systolic areas were acquired from the apical 4-chamber view.  The *right ventricular index of myocardial performance* (RIMP) was the isovolumic time divided by the ejecting time, which was evaluated in the same pulsed. The *isovolumic time* was computed by subtracting the ejecting time from the tricuspid closure time. All findings were summarized in Table 4.

**Table 4:** Changes of the recommended measures of right heart structure and function in 48 studied participants over the time

RV function indices	Before transplant	One week after transplant	One month after transplant	3 months after transplant	Mean square	F	*P*-value^*^
RV thickness (mm)	5.16 ± 0.99	5.21 ± 1.09	4.91 ± 1.31	5.1 ± 0.96	0.604	0.376	0.62
RV global longitudinal strain	−16.6 ± 5.8	−17.9 ± 5.0	−18.9 ± 5.54	−19.9 ± 4.8	107.2	3.85	0.024
RV free wall longitudinal strain	−21.2 ± 5.2	− 23.1 ± 5.0	−24.4 ± 5.3	−26.3 ± 11	493.7	6.74	< 0.001
RV basal diameter (mm)	33.3 ± 5.7	29.2 ± 4.1	25.2 ± 1.6	25.5 ± 4.0	82.3	4.78	0.007
RV mid cavity diameter (mm)	26.6 ± 4.9	26.5 ± 5.7	26.2 ± 4.4	23.6 ± 2.8	30.9	1.54	0.22
RV longitudinal diameter (mm)	61.9 ± 14.6	60.5 ± 15.2	54.5 ± 5.9	51.6 ± 3.8	342.5	2.06	0.13
RA major diameter (mm)	44.7 ± 7.2	44.1 ± 6.3	42.6 ± 5.5	40.6 ± 3.1	222.7	10.3	< 0.001
RA minor diameter (mm)	33.9 ± 6.4	32.7 ± 5.7	31.1 ± 4.0	28 ± 2.6	1.868	13.93	< 0.001
Myocardial performance index	55.2 ± 24.4	55.8 ± 18.2	55 ± 13.5	55.1 ± 21	1182.9	2.797	0.067
Fractional area change (%)	43.2 ± 8.3	44.8 ± 7.6	46.6 ± 9.2	48.1 ± 7.2	164.6	2.97	0.048
Tricuspid annular plane systolic excursion (mm)	21.8 ± 6.6	21.8 ± 5.0	22.2 ± 6.6	23.1 ± 4.9	9.77	0.345	0.74
Tissue velocity	12.9 ± 2.1	13.7 ± 3.2	13.9 ± 3.7	14.2 ± 3.9	9.91	0.92	0.40
PAP (mmHg)	32.7 ± 9.5	30.2 ± 6.2	27.7 ± 7.7	26.4 ± 5.9	386.8	6.52	0.001
RV E/e’	7.2 ± 1.15	6.5 ± 1.18	6.4 ± 2.5	6.3 ± 2.0	13	0.49	0.55
RV deceleration time	175.1 ± 61.5	176.8 ± 44.9	163.6 ± 36.9	169.1 ± 44.3	1700.4	0.746	0.49
TAPSE/PASP	0.78 ± 0.20	0.76 ± 0.20	0.86 ± 0.22	0.92 ± 0.24	0.259	5.94	0.002
RA volume index	23.39 ± 7	26.20 ± 7.8	26.39 ± 4.8	24.20 ± 4.8	187.2	15.2	0.000

*Adjusted for age, BMI, duration of dialysis, serum creatinine and phosphorus level

**Fig. 1:**
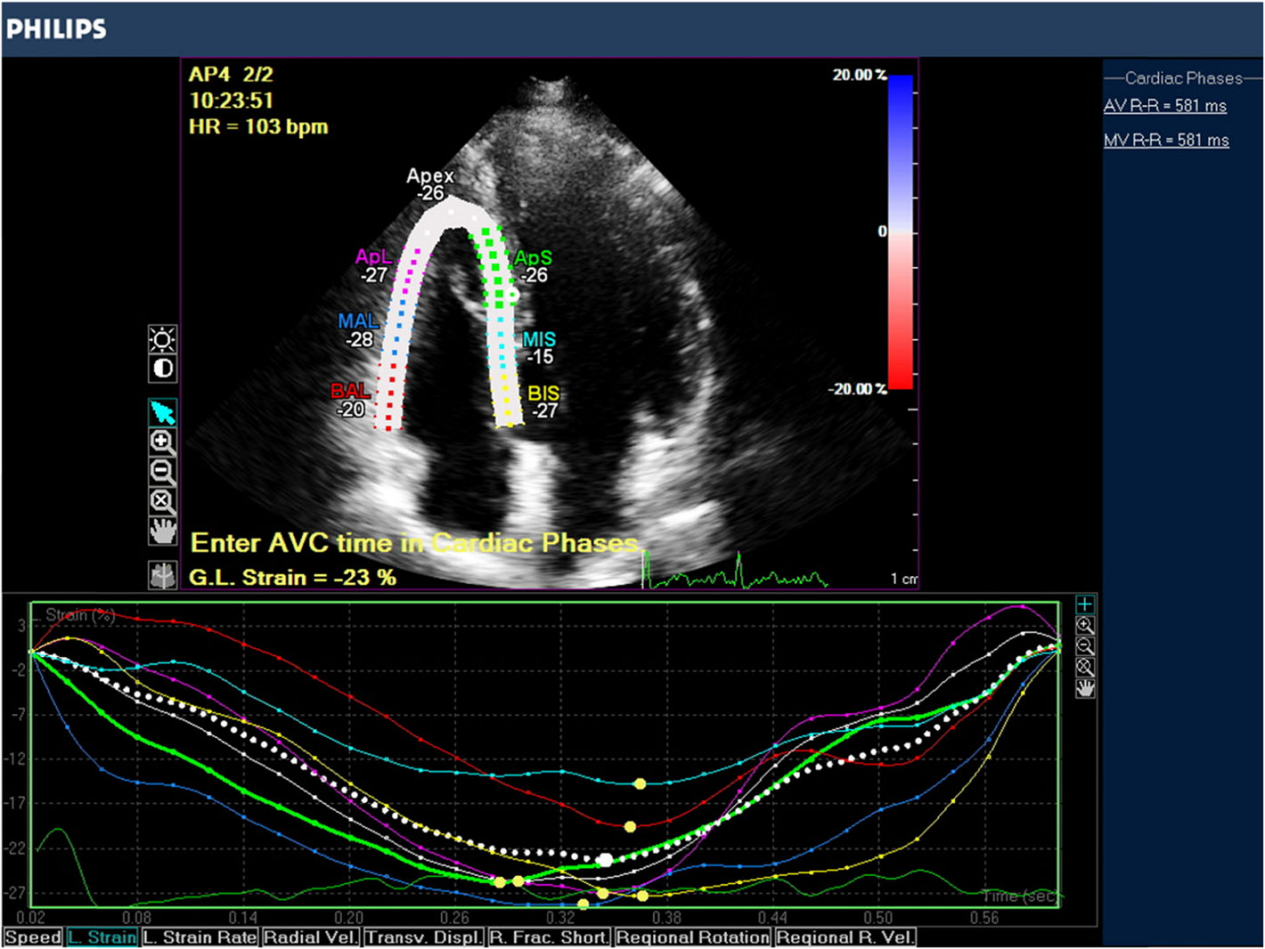
The assessment of RV systolic function by 2D STE longitudinal strain in an apical RV view

The strain analysis was done on an offline basis (Fig. 1). Generally, we traced a region of interest by point-and-click approach on the endocardium at end-diastole in RV from the RV-focused view. A second larger region of interest was further produced and manually fine-tuned near the epicardium. The region of interest was carefully adjusted using visual assessment to assure that every segment was tracked perfectly. The right ventricle was partitioned into 6 standard segments at 3 levels (i.e., the basal, middle, and apical levels), correspondingly generating 6 time-strain curves (Fig. 1). RV free wall longitudinal peak systolic strain (RV LPSS) was evaluated in the basal, midventricular, and apical segments of the RV free wall and calculated as the average of the 3 segments. RV global longitudinal peak systolic strain calculated automatically by machine. Longitudinal peak systolic strain is determined as the percentage of myocardial shortening relative to the original length and is conventionally presented as a negative value. Therefore, the more negative the value of RV LPSS and RV GLPSS, the more preserved is the shortening.

The echocardiographic findings were gathered and analyzed in a blind fashion.

#### Role of speckle tracking echocardiography

Myocardial strain imaging is a sensitive echocardiographic technique for identifying ventricular dysfunctions [6].

The measurement of myocardial strain by two-dimensional (2D) speckle tracking echocardiography (STE) has been demonstrated to be a feasible and sensitive quantitative technique for assessing left ventricular (LV) function for a variety of different cardiac diseases [15].

Several studies have revealed that RV longitudinal systolic strain measurements by STE may be a valuable method for assessing the RV function because it can provide prognostic data and is more reliable than conventional parameters [16–21].

Currently, a wide agreement regarding normal values is lacking and it seemed that it depends on the kind of machine and vendors as we can see in different articles [22].

According to Fine et al., the mean RV strain was − 26 ± 4% and the estimate of RV free wall strain measured using tissue Doppler imaging was − 27 ± 1% and with STE was − 27 ± 2% in subjects with normal echocardiograms and without cardiopulmonary disease or risk factors [6]. Furthermore, a recent analysis suggested that the RV free wall strain with the cutoff values from − 20% to − 21% seemed to be able to detect abnormal RV function [5].

### Statistical analysis

Continuous demographic and clinical data was demonstrated as mean and SD, and classified data was demonstrated as frequency and percentage.‌‌‌ Chi-square test or Fisher’s exact test were used to investigate the independency of the two categorical variables. To check the normality of the data, the Kolmogorov-Smirnov test was performed. The change shape in RV global longitudinal strain and RV free wall longitudinal strain versus time during the four measurements was tested using the fractional polynomial regression method. To estimate the regression line slope of main variables i.e. RV global longitudinal strain and RV free wall longitudinal strain, the data was reshaped from wide to long. After that, regression line slope was calculated by Stata, using 4 recorded measurements for each case. The Fracpoly model was also used to determine the type of relationship between the main variable and outcome. This model was used due to the continuous nature of mentioned variables, as well as the disadvantages of converting continuous to categorical data. Also, a General Linear Model (GLM), repeated measurement-Analysis of Variance (ANOVA) procedure was used to compare the changes occurring in structural and functional RV indices before and 1 week, 1 month and 3 months after successful kidney transplant. The assumption of sphericity of this analysis was performed using Mauchly’s Test of Sphericity. Since the assumption of sphericity had been violated for any of the above three variables, in this analysis, the comparisons were performed using the Greenhouse-Geisser test. The significance level in all tests was considered 0.05. Data analysis was performed using stata MP13.

## Results

This study was performed on 48 kidney transplant recipients including 28 men (58.3%) and 20 women (41.7%) with mean age of 43.3 ± 13.9 years. Of the whole participants in the study, 11 patients (22.9%) had diabetes, 31 patients (64.6%) had hypertension, and 4 patients (8.3%) were smokers. In terms of types of dialysis, 6 patients (12.5%) were categorized in pre-emptive kidney transplantation, 37 patients (77.1%) were treated with hemodialysis, and 5 patients had peritoneal dialysis. Moreover, in this study, kidney transplant rejection occurred in none of the participants under study. Other demographic and clinical data are reported in the following table.

Table 1 shows the basic and demographic characteristics of the participants according to their gender.

**Fig. 2:**
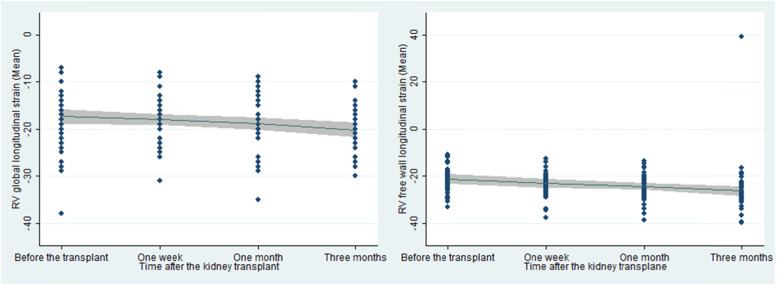
The linear relationship between RV global longitudinal strain and RV free longitudinal strain changes and time among the participants

The results of the above table showed there was no statistically significant difference between the women and men participating in the study regarding all the basic demographic and clinical variables except for the status of smoking and serum calcium levels. The results of the study on the shape of RV global longitudinal strain and RV free wall longitudinal strain changes, using the fractional polynomial models, showed that the form of the relationship was linear and they improved significantly over the time (*P* = 0.024, *P* < 0.001 respectively). This relationship is shown in Fig. 2.

Table 2 shows the results of the repeated measure ANCOVA to analyze the changes in the indexes related to right heart function after kidney transplantation among the participants over the time.

**Fig. 3:**
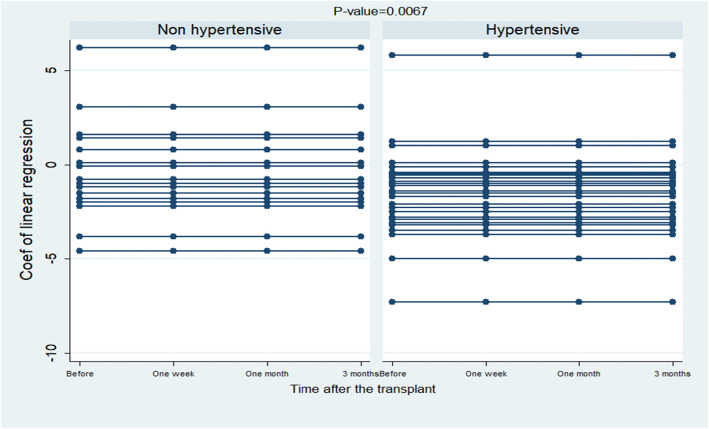
The regression line slope of RVGLS changes in hypertensive and non-hypertensive individuals

The results of the above table represented that kidney transplantation did not have significant effect on the RV mid cavity diameter, tissue velocity, Myocardial performance index, RV longitudinal diameter, and Tricuspid annular plane systolic excursion indices, but for other indices this effect was significant. On examining the statistical differences between the indicators that had a meaningful clinical and statistical downward or upward trend, the results of the analysis of variance with repeated measures showed there was no significant difference between diabetic and non-diabetic participants in terms of structural and functional indices of right heart during the study; thus, the two groups showed a decrease in this amount (*P* > 0.05). The results also depicted that a weak and non-significant negative correlation was observed between the age of the subjects in the study and RV global longitudinal strain (*P* = 0.24). On the differences between the mean slope of regression line of the GLS variable in hypertensive subjects (1.0 ± 0.2) and non-hypertensive subjects (0.36 ± 0.32), an independent t-test showed that between the two groups in terms of the improvement of RVGLS, there was a significant statistical difference (*P* = 0.0067) (Fig. 3).

**Fig. 4:**
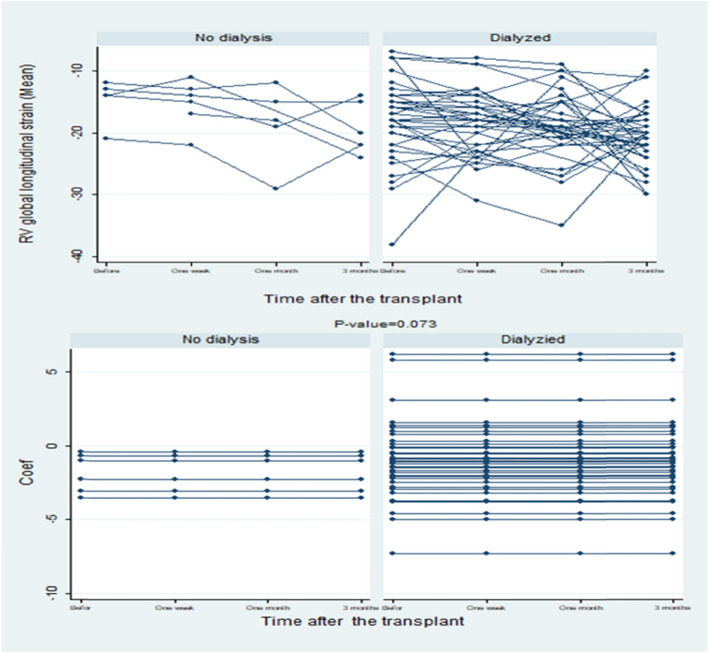
The slope of the regression line and the actual values of RV global longitudinal strain during the study

The results demonstrated that there was no statistically significant difference between the dialyzed and the non-dialyzed participants before the transplant in terms of the slope of the estimated regression line of RV global longitudinal strain (*P* = 0.6). Figure 4 depicts the regression line slope values and actual values in each of the participants, respectively.

**Fig. 5:**
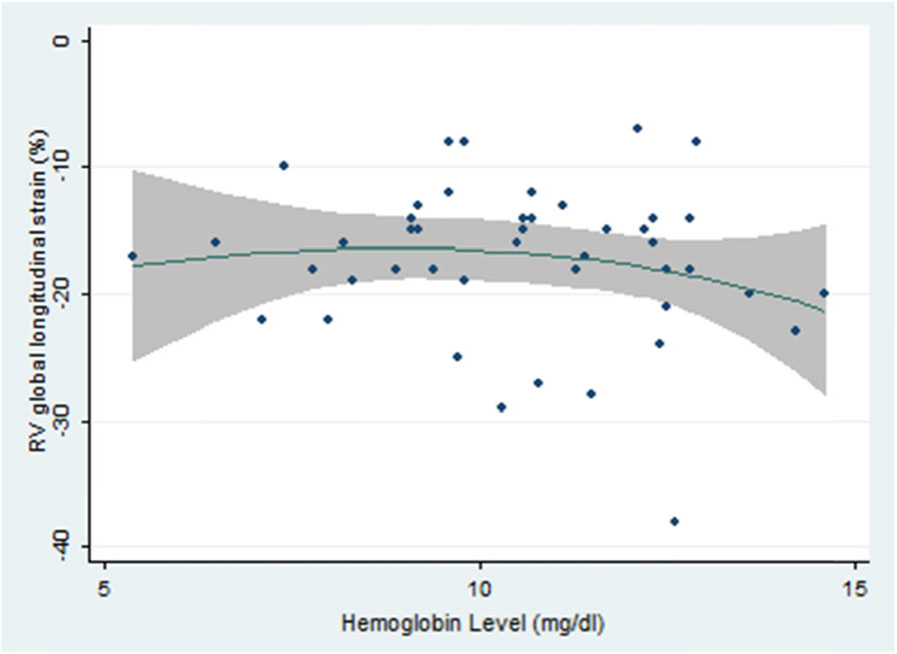
The relationship between the basal hemoglobin level and RV global longitudinal strain in individuals under study

In the correlation between basal hemoglobin levels before the kidney transplantation and basal GLS, the results represented that there was no statistically significant relationship between these two variables. In other words, as the number of hemoglobin units increased, the basal GLS level decreased by a factor of 0.4, but this decrease was not statistically significant (*P* = 0.35) (Fig. 5).

## Discussion

In this study, we sought to investigate whether kidney transplant could improve the RV function to be evaluated by the noninvasive method of 2D speckle tracking echocardiography and conventional methods such as TAPSE and RV FAC. In this prospective study, the results showed that there was a significant improvement in RV free wall (*P* < 0.001) and RV global strain (*p*-value = 0.024) and in PAP (*p*-value = 0.001) from baseline to 1 and 3 months following the kidney transplant. Also, it was found out RV diameter at the base (*p*-value = 0.007) and major and minor RA diameter and RA volume index decreased significantly (*p* < 0.001) after the successful kidney transplant.

Furthermore, it is important to clarify that all of the conventional methods such as MPI, TAPSE, Tissue Doppler velocity, and FAC correlated with the improvement, although the *P*-value were not statistically significant but were clinically valuable and significantthat this could be the result of low number cases. The amelioration of pulmonary hypertension after kidney transplant was another interesting subject that was mentioned in other studies before [23] and was in correlation with the decreased Tricuspid regurgitation severity which could be the result of the improvement afterload indices like LV filing pressure and LV diastolic function.

Ferrara et al. [24] evaluated 1168 healthy subject for providing TAPSE normal cut-offs. They found that TAPSE and S′ are more under effect RV pre-load echo-doppler indices such as LV cardiac output and RV basal dimension and less correlate with echo-doppler indices reflecting RV afterload such as PASP.

We also evaluated RV diastolic function by diastolic indices. There was no significant improvement in E/e’ and RV deceleration time after transplantation (*p* = 0.55 and *p* = 0.49 respectively); it could be due to low volume cases and short follow-ups.

It seems that RVLS associated with negative outcomes in the diseases affecting RV function [25, 26], so it could be used as a standard and reproducible method to evaluate RV function [22].

This was the first study to report and assess RV function by RV speckle tracking and compare it with conventional methods in patients with ESRD before and after kidney transplantation. RV function was improved even in the patients who were in the normal range before the transplantation. Therefore, it was asserted that RV speckle tracking could be a more sensitive index for the evaluation of RV function because in some cases in which the conventional values were normal, RV strain was reduced.

Many previous studies evaluated the effect of successful kidney transplant on LV mass, LV ejection fraction, and LV diastolic function, but RV indices as a neglected and important chamber was never evaluated; thus, in this study, we focused on the RV chamber in spite of measuring the LV indices. Souza et al. [27] prospectively evaluated 40 patients with chronic kidney diseases immediately before and 1 month, three, and 6 months after kidney transplantation. They discovered that the basal ejection fraction and mean E/e’ were associated with reduced LV mass index after kidney transplantation. The LV mass index at the baseline, female sex, and the decrease in serum phosphorus were associated with a reduction in the mean E/e’ ratio after kidney transplantation.

Salerno et al. [28] evaluated 104 patients underwent kidney transplant by echocardiography being repeated every year for 3 years and during this process all the patients kept on the primary therapy. They observed that after kidney transplant, there was a reduction of LVH comparing to the pretransplant echocardiographic evaluations. The two immunosuppressive regimen, low dose calcineurine inhibitor (CNI) + everolimus (EVE), or CNI+ mycophenilate mofetil (MMF) did not affect the evolution of post-transplant LVH.

Casas-Aparicio et al. [19] evaluated 35 patients with CKD who had successful kidney transplant. They measured LV function and pulmonary arterial pressure by echocardiography before and 1 year after the transplant and found that LV diameters, wall thickness, and pulmonary arterial pressure were significantly decreased after the transplant.

Hatem Abdel Rahman Helmy Ali et al. [20] revealed that the 2-D longitudinal speckle tracking could distinguish early ventricular (left and right) systolic dysfunctions in patients with uremia in the presence of normal systolic function by conventional methods. Their study’s population included 24 newly diagnosed uremic patients, 25 hemodialysis patients, and 20 healthy individuals. They also found out RV and LV longitudinal strains were significantly lower in newly diagnosed uremic patients and hemodialysis patients compared to healthy subjects. In addition, they declared that in the hemodialysis group, RV longitudinal strain was significantly lower than that of the non-hemodialysis group; hence, hemodialysis by itself was regarded as a risk factor for the RV dysfunction independently. As it was mentioned earlier, the severity of improvement was significantly better in non-hypertensive patients compared to the hypertensive ones after kidney transplant. Thus, this is in line with the results of the study by Hatem Abdel Rahman Helmy Ali et al. [20] declaring there is a relationship between RV longitudinal strain and hypertension and LV strain.

In the absence of larger population-based registry data and multicenter studies, we tried to compare each patient with him/herself and perform all the measurements with one machine and one expert operator.

In clinical practice, two-dimensional longitudinal systolic measurement may be more useful for the RV than the LV; due to the fact that the RV requires analysis from only a unique apical view, but LV strain includes analysis from three apical views. Besides, since the dominance of RV is in the longitudinal direction, longitudinal strain leads to a graded approach to the RV systolic dysfunction, ranging from mild to severe impairment [6].

## Limitations

This study had several limitations. First, the strain algorithms used in this study were validated for LV strain and were not designed for assessing RV strain, while the strain algorithms were not chamber-specific.

Second, as it was a single-center study, the number of patients were small and follow-ups’ duration was short; thus, apparently it is needed to carry out a multi-center study with high volume cases and longer follow-ups.

Third, although there were multiple ultrasound platforms available, we only evaluated all data by only one vendor and platform (Philips IE33). According to Il’Giovine et al. [22], different global RV longitudinal strain values are not statistically significant among vendor-specific software (VSS) packages (*P* value≥.05).

Fourth, the normal range can be subjective because there are no established cutoffs for such studies.

## Conclusion

Sometimes, the impaired RV myocardial function in ESDR patients cannot be detected by conventional methods but can be detected by more sensitive newer methods such as 2D speckle tracking echocardiography. Additionally, it was depicted that successful kidney transplantation could improve RV function regardless of other risk factors except for hypertension and immunosuppressive regime.

Consequently, it is reemphasized that kidney transplantation is the treatment choice for ESRD patients who are eligible for that.

## Data Availability

As you request, it will be evaluated and if possible, the data will be available for you.

## Abbreviations

ESRD0: End stage renal disease;
2D: Two dimensional;
CKD: Chronic kidney diseases;
GLS: Global longitudinal strain;
RV: Right ventricle;
LV: Left ventricle;
RA: Right atrium
PASP: Pulmonary artery systolic pressure
FAC: Fractional area change
TAPSE: Tricuspid annular plane systolic excursion
MPI: Myocardial performance index
STE: Speckle tracking echocardiography
Hb: Hemoglobin
